# Further evidence for increased macrophage migration inhibitory factor expression in prostate cancer

**DOI:** 10.1186/1471-2407-5-73

**Published:** 2005-07-06

**Authors:** Katherine L Meyer-Siegler, Kenneth A Iczkowski, Pedro L Vera

**Affiliations:** 1 Research and Development Service (151), Bay Pines Veterans' Administration Medical Center, Bay Pines, FL 33744, USA; 2 Department of Surgery, University of South Florida, Tampa, FL 33612, USA; 3 Department of Pathology, Malcom Randall Veterans' Administration Medical Center, Gainesville, FL 32608, USA; 4 Department of Pathology, Immunology, and Laboratory Medicine, University of Florida College of Medicine, Gainesville, FL 32610, USA

## Abstract

**Background:**

Macrophage migration inhibitory factor (MIF) is a cytokine associated with prostate cancer, based on histologic evidence and circulating (serum) levels.

Recent studies from another laboratory failed to document these results. This study's aims were to extend and confirm our previous data, as well as to define possible mechanisms for the discrepant results. Additional aims were to examine MIF expression, as well as the location of MIF's receptor, CD74, in human prostatic adenocarcinoma compared to matched benign prostate.

**Methods:**

MIF amounts were determined in random serum samples remaining following routine PSA screening by ELISA. Native, denaturing and reducing polyacrylamide gels and Western blot analyses determined the MIF form in serum. Prostate tissue arrays were processed for MIF *in situ *hybridization and immunohistochemistry for MIF and CD74. MIF released into culture medium from normal epithelial, LNCaP and PC-3 cells was detected by Western blot analysis.

**Results:**

Median serum MIF amounts were significantly elevated in prostate cancer patients (5.87 ± 3.91 ng/ml; ± interquartile range; n = 115) compared with patients with no documented diagnosis of prostate cancer (2.19 ± 2.65 ng/ml; n = 158). ELISA diluent reagents that included bovine serum albumin (BSA) significantly reduced MIF serum detection (p < 0.01). MIF mRNA was localized to prostatic epithelium in all samples, but cancer showed statistically greater MIF expression. MIF and its receptor (CD74) were localized to prostatic epithelium. Increased secreted MIF was detected in culture medium from prostate cancer cell lines (LNCaP and PC-3).

**Conclusion:**

Increased serum MIF was associated with prostate cancer. Diluent reagents that included BSA resulted in MIF serum immunoassay interference. In addition, significant amounts of complexed MIF (180 kDa under denaturing conditions by Western blot) found in the serum do not bind to the MIF capture antibody. Increased MIF mRNA expression was observed in prostatic adenocarcinoma compared to benign tissue from matched samples, supporting our earlier finding of increased MIF gene expression in prostate cancer.

## Background

Macrophage migration inhibitory factor (MIF) is a cytokine initially isolated based upon its ability to halt the *in vitro *random migration of macrophages [1,2]. Subsequently, it was defined as a proinflammatory cytokine with a key regulatory role in inflammation and both specific and nonspecific immunity [3]. Currently MIF is recognized as being constitutively expressed in various cell types, able to manifest itself as a cytokine, hormone or enzyme, and as a mediator of many pathologic conditions [4].

As a proinflammatory cytokine, MIF counter-regulates the effects of glucocorticoids and stimulates the secretion of other cytokines such as tumor necrosis factor (TNF)-α and interleukin (IL)-1β [3], thus assuming a role in the pathogenesis of inflammatory, immune diseases and cancer [5-7]. MIF is directly associated with the growth of lymphoma, melanoma, and colon cancer [8]. Indications of MIF's key role in tumor progression derive from studies where treatments with either anti-MIF immunoglobulin therapy and/or MIF antisense oligonucleotide confer anti-tumor activity [9,10]. Although MIF is associated with cancer angiogenesis, progression, and metastasis, the exact mechanism of this cytokine's action is uncertain since a receptor for MIF has only recently been identified as the cell surface form of the invariant chain (CD74) [11].

This laboratory was first in localizing MIF within prostatic epithelium and in establishing that MIF is consistently increased within both prostate tissue and serum in prostate cancer patients [7,12,13]. The expression of CD74 in the human prostate or its association with prostate cancer has not been investigated, but if MIF affects prostate cancer cell growth, then it is predicted that MIF's receptor should be localized within prostate tissue. Our recent paper established that patients with prostate cancer had elevated serum MIF levels [13]. However, a study from Michael et al. failed to confirm these findings [14]. This discrepancy is worth investigating since establishing the exact role of MIF in prostate cancer may prove useful as a diagnostic or prognostic indicator (in addition to the well-established standard, prostate specific antigen – PSA) for this important disease entity.

The objectives of this study are: (1) to confirm and extend our previous serum findings by analyzing serum samples remaining following routine serum PSA analysis from both prostate cancer and non-prostate cancer patients; (2) to identify potential confounds within the ELISA protocol that may account for the contradictory findings reported [14]; (3) to localize, quantify, and compare MIF mRNA and protein amounts in matched cancer and benign biopsy samples using commercially available tissue arrays; (4) to localize the MIF receptor, CD74, within prostate tissue and (5) to compare MIF secretion by normal prostate epithelial cells and prostate cancer cells *in vitro*.

## Methods

Approval for all facets of this study was obtained from the Bay Pines Veterans' Administration Medical Center (VAMC) Institutional Review Board and the study protocols comply with the Helsinki Declaration. The serum samples were obtained with waiver of informed consent under section 46.116(d) of the Department of Health and Human Services human subject regulations at 45 CFR 46.

### Comparison of MIF serum amounts in prostate cancer and non-cancer patients

Random serum samples remaining following routine PSA evaluation prior to being discarded were collected (n = 212) and stored at -80°C prior to analysis. Clinical PSA values for the serum samples (determined by the radioimmunoassay laboratory at the Bay Pines VAMC) and documentation of prostate cancer were obtained through analysis of computerized records. Normal patients were defined as those without documented prostate cancer and with serum PSA values at or below 2 ng/ml (n = 158).

Samples were assayed for MIF using the protocol described by the manufacturer (DuoSet ELISA, R&D Systems, Minneapolis, MN) with the following modifications: Milk diluent, (500-92-01, Kirkegaard and Perry Laboratories, Gaithersburg, MD) was used at a 1:20 dilution as the blocking agent and serum diluent. Our previously published data set of MIF serum levels in prostate CaP patients was also included for comparison (n = 61) [13]. Determined values for serum PSA and MIF were not normally distributed, therefore differences in serum PSA and MIF amounts were assessed by Kruskall-Wallis ANOVA followed by post-hoc tests (Dunn's multiple comparison test). Significance was defined as p < 0.05. Data are reported as median ± interquartile range. Statistical analysis was performed using Prism statistical analysis software version 4.02 (GraphPad Software, Inc., San Diego, CA).

### Comparison of different ELISA protocols on MIF serum determination

Michael et al. used the MIF DuoSet ELISA system to determine serum MIF [14]. However, there were several deviations from the manufacturer's protocol and from our own protocol, principally the use of bovine serum albumin (BSA) as a blocking agent and serum diluent. Therefore, to test whether differences in ELISA protocols result in discrepant findings, in a separate experiment 10 random serum samples (n = 5 CaP; n = 5 non-CaP) were assayed in parallel using either milk diluent (our established protocol in agreement with the manufacturer's suggestions) or PBS with 1% BSA (as reported by Michael, et al. [14] as the blocking agent and serum diluent). All other steps in the protocol were as described by the manufacturer (R&D Systems). Comparisons between different ELISA protocols were evaluated using two-tailed unpaired nonparametric t-tests (Mann-Whitney). Data are reported as median ± interquartile range.

### MIF forms in human serum

The discrepancy between these ELISA data could be attributed to blockage of the monoclonal capture antibody (mAb) epitope due to MIF interaction with other serum proteins. Changes in the dilution buffer composition may alter these interactions resulting in unmasking of capture antibody binding sites. To address this possibility, serum proteins (diluted 1:20 with PBS) were separated by native, denaturing and reducing polyacrylamide gel electrophoresis following the manufacturer's protocols (4–20% Tris-glycine or 4–12 % NuPAGE Bis-Tris gels, Invitrogen, Carlsbad, CA) and transferred to Invitrolon (Invitrogen). Reducing conditions included addition of anti-oxidant (Invitrogen) to running buffer and transfer buffer to prevent oxidation of reduced cysteine, methionine and tryptophan residues. Blots were incubated overnight with a biotinylated MIF specific, affinity purified, antibody (BAF289, R&D Systems) at 1:1000 dilution overnight at 4°C as described previously [15].

Serum MIF was immunoprecipitated using agarose-immobilized ELISA capture mAb (R&D Systems antibody; Profound co-immunoprecipitation system, Pierce, Rockford, IL) to characterize further mAb MIF binding forms. For immunoprecipitation studies, 2 ml of serum was first cleared of contaminating IgG by passage through a Protein G column (Pierce) and 1 ml PBS fractions collected. The eluted fraction with the highest protein content was batch incubated with 200 μl immobilized capture mAb (200 μg MIF mAb per 100 μl of packed resin) overnight with continuous rocking at 4°C. Following overnight incubation the agarose resin was transferred to a spin column, proteins that did not stick were collected, and the column washed with 5, 1 ml PBS washes. The bound proteins were eluted and proteins in all fractions separated under denaturing and reducing conditions on 4–12% NuPAGE Bis-Tris gels, and then transferred to Invitrolon membranes following the manufacturer's protocols (Invitrogen). In some instances, fractions with low total protein content were first concentrated 800 fold (Eppendorf vacuum concentrator, Westbury, NY).

In all instances, MIF Western blots were performed using a polyclonal biotinylated antibody at 1:1000 dilution followed by strepavidin-horseradish peroxidase conjugate used at a 1:500 dilution (R&D Systems). Antibody specific bands were detected using a chemiluminescent substrate (Pierce) and bands quantified as described previously [15]. Data are expressed as net intensity values normalized to the staining intensity of a recombinant MIF standard (7.5 ng).

### Determination of MIF and CD74 in matched prostate cancer and benign samples

Tissue array slides were obtained from Ambion (Low density; Austin, TX) and used for both *in situ *hybridization (MIF only) and immunohistochemistry (IHC; MIF and CD74). The slides contained paired samples of prostatic adenocarcinoma and matched benign specimens from the same patient. The present investigation was restricted to comparison of expression in matched benign versus cancer samples. Samples that could not be matched (e.g. missing section on the slide) were not included in the analysis.

#### *In situ *hybridization

RNase inhibitor (Protect-RNA, Sigma, St. Louis, MO) was included in all aqueous solutions. Tissue sections were dewaxed and rehydrated through ethanol, then treated with Proteinase K for 30 min at 37°C and endogenous peroxidase quenched by 10 min incubation in 3% hydrogen peroxide. MIF biotinylated probes were prepared by reverse transcription, followed by amplification of a 254 base pair (bp) MIF fragment [13]. T7 RNA polymerase promoters were ligated to the PCR product (Ambion) and single stranded RNA oligonucleotide probes produced by *in vitro *T7 transcription (Promega, Madison, WI) with incorporation of biotinylated-dUTP. Hybridized probes were detected with avidin-peroxidase (ISH-B1, Sigma). Negative controls consisted of hybridization of tissue arrays with sense biotinylated probes and hybridization of test prostatic tissue sections without probe addition. A poly-d(T) biotinylated probe was used as a positive control in test prostatic tissue sections. Tissue arrays were not counterstained.

#### Immunohistochemistry

Paraffin sections (4 μm) of formalin-fixed tissue arrays were dewaxed and processed for antigen retrieval (citrate buffer, pH 6.0; microwave 600 W to boiling, 2 min rest, and repeat cycle 10 times for a total time of 20 min). Sections were treated with 3% H_2_0_2 _for 2 min to quench endogenous peroxidase, preincubated in normal donkey serum (3%; 30 min) and then exposed to MIF biotinylated polyclonal antibody (BAF289, R&D Systems; 1:1000 in PBS, 0.3% Triton X-100) or CD74 antibody (sc-5438, Santa Cruz Biotechnology, Santa Cruz, CA; 1:400 in PBS, 0.3% Triton X-100) overnight at 4°C.

Previous experiments demonstrated the specificity of MIF labeling of prostatic tissues [12]. Specificity of CD74 immunostaining was confirmed on a separate set of prostatic tissue slides by omitting the primary antiserum (data not shown). Bound primary antibody was detected using biotinylated secondary antibody (Vector Laboratories, Burlingame, CA) and diamidinobenzidine (DAB) according to the manufacturer's protocol. The slides were counterstained with hematoxylin, dehydrated, cleared in xylene and coverslipped.

All tissue sections were scored for the location and intensity of staining (0 = no staining detected through 3 = very strong staining). MIF mRNA and MIF/CD74 immunostaining was examined in matched tissue samples. Differences were assessed in epithelial MIF staining intensity in matched versus adenocarcinoma samples using paired, two-tailed t-tests.

### Determination of MIF expression in normal and prostate cancer epithelial cells *in vitro*

Normal prostate epithelial cells (PrEC, Cambrex, Walkersville, MD) and prostate cancer cell lines (LNCaP and PC-3; American Type Culture Collection, Manassas, VA) were cultured until 80% confluent according to previously described protocols [16]. Media were exchanged for serum free PrEGM (Cambrex) and following 48 h incubation, samples of culture medium containing equal total protein concentrations were analyzed by denaturing Western blot as described above. The resultant protein bands from three separate Western blots from each cell line were quantified by digital imaging (Kodak, Rochester, NY). Data are expressed as fold change compared to normal cells and differences assessed by unpaired, two-tailed t-tests.

## Results

### Comparison of MIF serum amounts in prostate cancer and non-cancer patients

**Figure 1:**
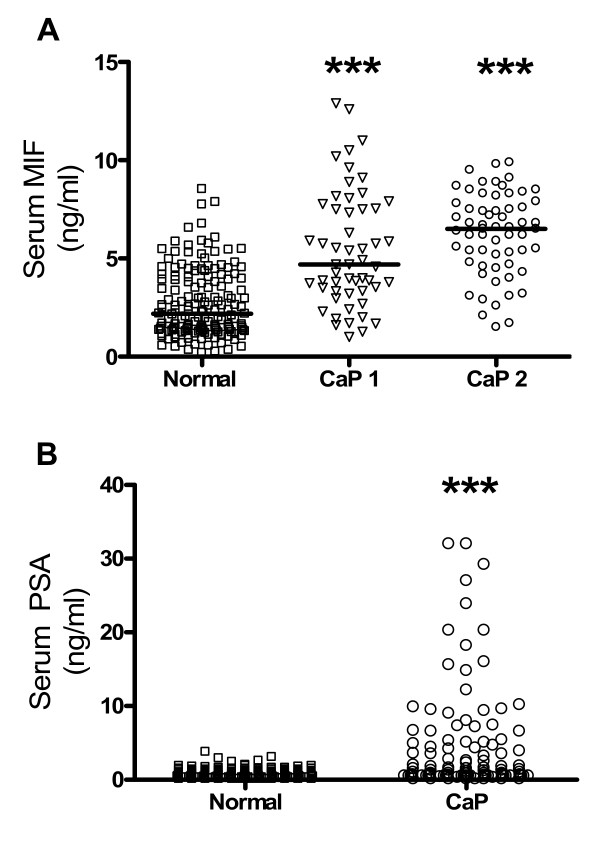
**Scatter plots of PSA and MIF in random serum samples obtained following routine PSA testing**. Median values for each group are represented by horizontal lines. Diagnosis of prostate cancer was determined by chart review. *** = p < 0.001 A) Serum MIF – All data points were mean values of duplicate ELISA analyses. B) Serum PSA – All data were obtained from computerized records of clinical laboratory analysis.

We determined MIF serum levels in normal (non-CaP) and prostate cancer (CaP) patients using milk based buffer as the ELISA blocking agent and sample diluent. Using this protocol, the median MIF serum level in CaP patients (Fig. 1, CaP 1; 4.70 ± 4.32 ng/ml; ± interquartile range) was significantly greater than that observed in non-CaP patients (2.19 ± 2.65 ng/ml; Fig 1; <0.001). Thus, we confirm our previous observation of increased MIF serum levels in CaP patients [13].

Furthermore, we compared our previously analyzed cancer patients with our current group to detect any possible differences. Our previous CaP group had a median MIF serum level of 6.50 ± 3.10 ng/ml (Fig. 1, CaP 2) that was not statistically different from our current CaP group (Fig. 1, CaP 1; p = 0.495) although it was still significantly elevated when compared to the control group (Fig 1; p < 0.001).

Since no significant differences were observed between the two cancer groups, we have pooled the serum MIF levels to result in a combined prostate CaP group. The controls from our first study were from serum samples used for thyroid hormone analysis (n = 14) and were not combined with this normal group since the corresponding PSA values are unknown [13]. Thus, our data show that CaP patients have a significantly higher MIF serum level (5.87 ± 3.91 ng/ml, n = 115) when compared to non-cancer patients (2.19 ± 2.65 ng/ml, n = 158) and this difference is statistically significant (p < 0.0001). All serum samples were obtained from patients at least 15 months post-CaP diagnosis and median PSA serum amounts were significantly increased in the CaP (1.20 ± 4.60 ng/ml, ± interquartile range, n = 115) patients (Fig. 1B, p < 0.001) suggesting that this group of patients had recurrent or metastatic prostate cancer.

### Comparison of different protocols on MIF serum determination

**Figure 2:**
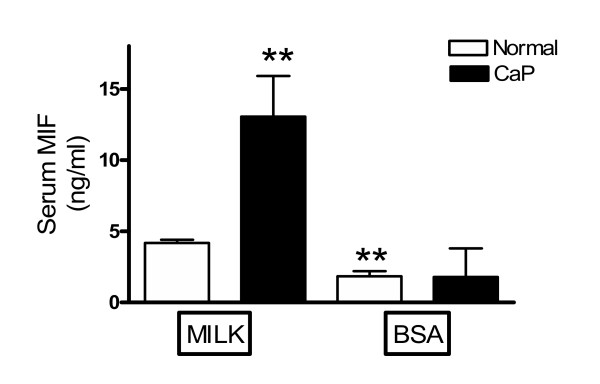
**Comparison of Serum MIF amounts determined by different ELISA conditions**. A subset of random serum samples were from pulled from our serum bank from patients with no documented diagnosis of prostate cancer (n = 5) and from patients with CaP (n = 5). The serum MIF amounts were determined in parallel using either a milk based serum blocking/diluent solution or phosphate buffered saline pH 7.4 with 1% bovine serum albumin. Data are expressed as median ± interquartile range, ** = p < 0.01, when compared to Milk normal serum samples.

We noted several differences between the MIF ELISA protocol of Michael et al. [14] and our serum MIF ELISA protocol. Since MIF's interactions with albumin subfractions have been described [17], we tested the possibility that use of BSA in the MIF ELISA protocol would interfere in the MIF ELISA assay. Thus, we compared a subset of serum samples from prostate cancer patients (n = 5) and from patients with no documented prostate cancer (n = 5), which were assayed in parallel using either milk based buffer (our protocol) or PBS with 1% BSA (Michael et al. [14] protocol) as the serum diluent and blocking agent. Serum MIF levels were significantly increased, even in this small sample, when we used the milk-diluent based MIF ELISA (our protocol), with non-CaP having 4.18 ± 0.99 ng/ml compared to prostate cancer patients that had 13.06 ± 6.15 ng/ml of MIF in the serum (Fig. 2). However, the same samples, when run using the BSA diluent (as reported by Michael et al. [14]) showed no significant difference between the two groups (Fig. 2), in agreement with their findings. Moreover, when the same set of normal sera was compared using milk-diluent versus BSA diluent, it was observed that the BSA protocol resulted in a statistically significant lower MIF serum level (1.84 ± 0.73 for BSA compared to 4.18 ± 0.99 for milk). These data demonstrate that addition of BSA to the MIF serum ELISA protocol results in not only interference with the ability to detect an increase in MIF serum levels, but it also decreases the ability of the assay to detect MIF (as evidenced by the significantly lower MIF serum levels observed in patients without prostate cancer with the BSA protocol).

### MIF forms in human serum

**Figure 3:**
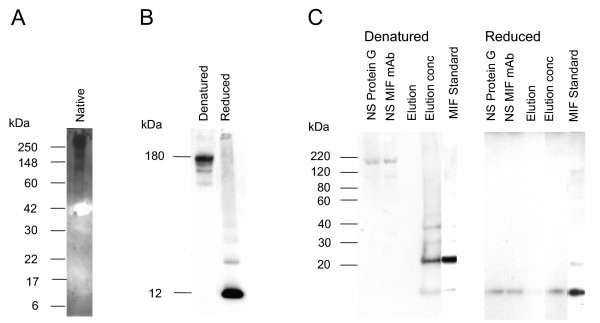
**MIF Western blot of serum proteins**. A. Native serum MIF – Serum was diluted 1:20 in PBS and proteins were separated under native conditions then analyzed for MIF by Western blot analysis. Under native conditions, a broad band between 150 – 500 kDa was detected. B. Denatured and reduced serum MIF – Serum was diluted as in A and proteins separated under denaturing or reducing conditions. Denaturing conditions resulted in a single prominent band at 180 kDa and some minor bands between 80 and 120 kDa. Under reducing conditions, a single prominent 12-kDa band and a minor band at 24 kDa were observed. C. mAb Affinity purified serum MIF – MIF containing serum proteins were purified by sequential affinity chromatography through Protein G agarose, followed by MIF mAb affinity chromatography. Nonsticking proteins as well as elution fractions were separated by NuPAGE gel electrophoresis under denaturing or reducing conditions, transferred to PVDF and MIF containing bands identified using a polyclonal antibody (R&D Systems, 1:1000 dilution). Lanes left to right: NS Protein G – non-sticking fraction from Protein G column; NS MIF mAb – non-sticking fraction from MIF monoclonal antibody column; Elution – third elution fraction; Elution conc – third elution fraction concentrated 800 fold; MIF standard – 7.5 ng of recombinant MIF protein. Numbers and lines to the left of each Western blot indicate location of molecular weight markers.

The success of antigen capture ELISA reactions is dependent upon accessibility of the epitope. Detecting antigen bound to endogenous proteins in serum is often difficult because of epitope masking or sensitivity problems. MIF's association with other proteins (either as carrier proteins or activators) has not received much attention although it may shed light on the mechanism of action of this complex molecule. While many studies have detected serum MIF, to date there is no information on the MIF form(s) in human serum. MIF Western blots under native conditions showed only a large high molecular weight band in the range of 150 to 500 kDa. (Fig. 3A). No monomeric, dimeric or trimeric MIF was observed. Under denaturing conditions, a single MIF immunoreactive band was detected (180 kDa). Again no monomeric, dimeric or trimeric MIF was observed. Under reducing conditions only a single 12 kDa MIF band was detected, suggesting that this MIF-protein complex is either held by covalent bonds or that the reducing conditions alters the complex conformation allowing the MIF to be released. The same banding pattern was observed using a well characterized MIF monoclonal antibody (III.D.9), suggesting that this banding pattern was not the result of antibody cross reactivity or non-specific binding (data not shown).

The ability of the ELISA mAb to detect all the MIF in serum samples was analyzed by affinity chromatography with the ELISA capture antibody followed by Western blotting (Fig. 3C). Following removal of immunoglobulin by Protein G chromatography, serum MIF was found in a 180 kDa complex (Fig. 3C, Denatured, NS Protein G). The MIF capture monoclonal antibody (Fig. 3C, Denatured, NS MIF mAb) did not recognize this 180 kDa MIF form. Affinity captured MIF was not detectable by Western blot without concentration of the eluted fraction (Fig. 3C, Denatured, Elution, Elution conc). The concentrated (800 fold) mAb captured MIF was separated into four bands of less than 40 kDa (12, 24, 30 and 36) with the 24 kDa band being the predominant form detected. The serum concentration of the180 kDa MIF form was 220 ng/ml, while the concentration of the 24 kDa band was 1.5 ng/ml as determined by normalization of band intensities to the MIF recombinant protein. Therefore, the MIF ELISA appears to detect less than 1% of the total serum MIF detected by Western blot.

The 180 kDa MIF complex was disrupted by reducing agents as only the 12 kDa monomeric MIF form was detected with addition of dithiolthreitol to the sample (Fig. 3C, Reduced). Under reducing conditions the MIF captured by the monoclonal antibody could not be detected without concentration (Fig. 3C, Reduced, Elution, Elution conc).

### Determination of MIF and CD74 in matched prostate cancer and benign samples

Only matched samples of benign and adenocarcinoma tissue were included in the final analyses (MIF *in situ*, n = 20; MIF IHC analysis, n = 20; CD74 IHC, n = 18). The patient ages ranged from 59 to 94 years, with a median of 72.5 years. All tumors were TNM classification T1a or T1b.

#### MIF mRNA in the prostate

**Figure 4:**
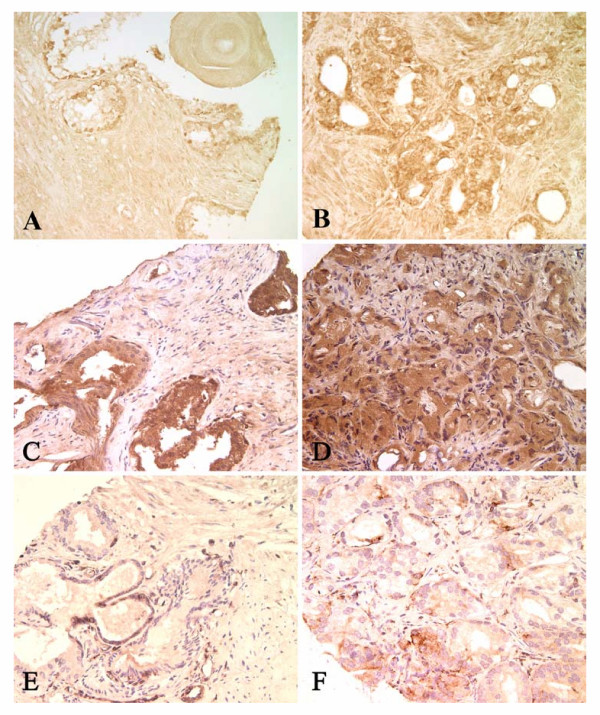
**Matched prostatic tissue staining**. All panels are 400 × total magnification. A) *In situ *hybridization – MIF in benign tissue B) *In situ *hybridization – MIF in matched cancer tissue C) Immunohistochemistry – MIF in benign tissue D) Immunohistochemistry – MIF in matched cancer tissue E) Immunohistochemistry – CD74 in benign tissue F) Immunohistochemistry – CD74 in matched cancer tissue

**Figure 5:**
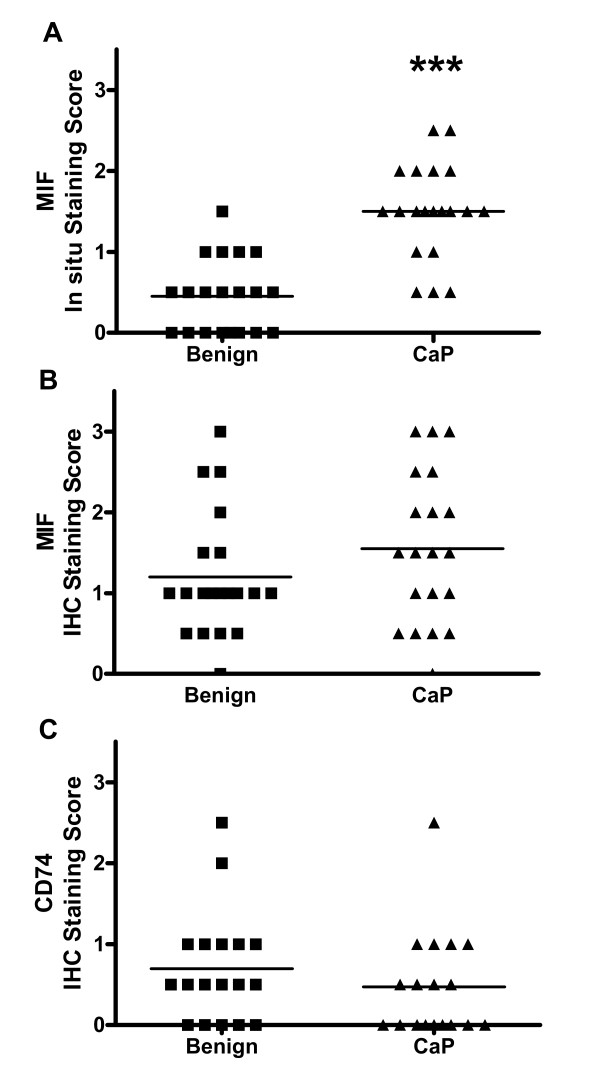
**MIF *in situ *hybridization and IHC staining intensity in prostatic tissue arrays**. Mean values for each group are represented by horizontal lines. Values are expressed as mean ± standard error. A) MIF *in situ *– intensity of epithelial cell staining of matched benign and tumor regions of prostate biopsies from prostate cancer patients (n = 20). B) MIF IHC – intensity of epithelial cell staining of matched benign and tumor regions of prostate biopsies from prostate cancer patients (n = 20). C) CD74 IHC – intensity of epithelial cell staining of matched benign and tumor regions of prostate biopsies from prostate cancer patients (n = 18). (*** = p < 0.001).

*In situ *hybridization of 20 matched samples of benign and cancerous prostate tissue revealed minimal MIF mRNA in the epithelium of benign samples, which was restricted to the cytoplasm (Fig. 4A). Increased MIF mRNA was detected in the matched cancer samples (Fig. 4B), which was localized to the cytoplasm with some evidence of nuclear or perinuclear localization of the signal. The mean *in situ *hybridization intensity of prostate cancer was 1.5, significantly higher than in matched benign tissues whose mean score was 0.5 (Fig. 5A; p < 0.001).

#### MIF protein location and intensity in the prostate

MIF immunostaining was readily observed in both cancerous and benign prostate in the 21 matched samples (Fig. 4C and 4D). MIF protein was localized predominantly within the epithelium in both benign prostate and cancer (Figs. 4C and 4D). The mean staining intensity within the epithelium in benign samples was 1.4, while that of the epithelium in adenocarcinoma was 1.5 (Fig. 5B). Stromal staining was evident in both benign and adenocarcinoma matched samples with no significant difference in the mean staining score (data not shown).

#### CD74 location and intensity in the prostate

Weak CD74 immunostaining was detected in both benign and prostate cancer tissues (Fig. 4E and 4F). The staining was patchy and localized to epithelial cells. There was no significant difference noted in staining intensity when comparing matched benign (mean intensity score = 0.6, Fig. 5C) and prostate cancer specimens (mean intensity score = 0.4).

### Determination of MIF expression in normal and prostate cancer epithelial cells *in vitro*

**Figure 6:**
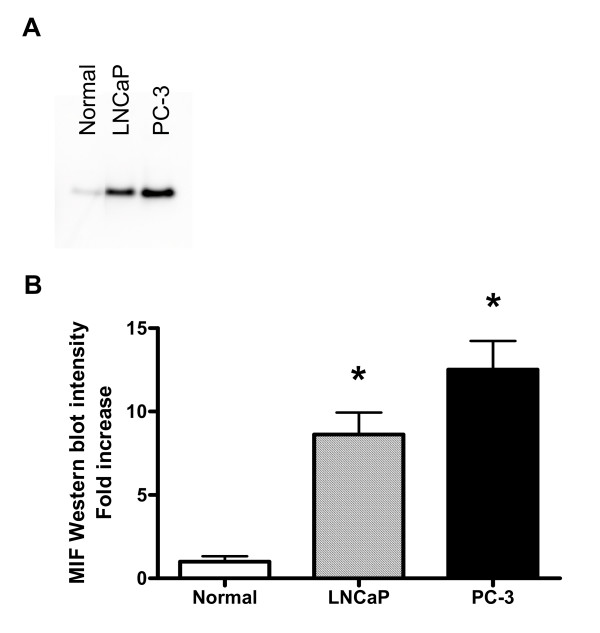
**MIF secreted by *in vitro *prostate cells**. A) Western blot of 48 h culture medium from 80% confluent normal, LNCaP and PC-3 prostate cells. B) MIF bands were quantified by digital imaging and are expressed as fold increase compared to normal. Data are presented as the mean ± standard error of three separate Western blots. (* = p < 0.05).

Western blotting of cell culture medium from prostate epithelial cells showed that all cell lines secreted only 12 kDa MIF into the PrEGM serum-free culture media (Fig. 6A). However, prostate cancer cells (LNCaP and PC-3) showed significantly greater MIF secretion when compared to the normal cell line (Fig. 6A). Analysis of the resulting band intensities documents an 8 to 12 fold increase in MIF amounts secreted by prostate cancer cell lines compared with normal prostate cells (Fig. 6B).

## Discussion

Recently attention has been focused on the possibility that epithelial cell injury as a result of chronic inflammation plays an important role in prostate carcinogenesis [18]. Thus, we proposed that MIF, a proinflammatory cytokine constitutively expressed by the prostatic epithelium and upregulated in prostate cancer, plays an important role in this disease entity [7]. Our present findings confirm our hypothesis since: 1) increased MIF serum levels were observed in an additional group of prostate cancer patients when compared to patients with no documented prostate cancer; 2) MIF mRNA was increased in prostate cancer tissue suggesting increased MIF synthesis in prostate cancer; 3) CD74, recently described as a receptor for MIF[11], is localized to prostatic epithelia; 4) prostatic epithelial cells *in vitro *secrete MIF, with much greater secretion observed in prostate cancer cells. Moreover, increased MIF expression in prostate cancer has been independently documented by other laboratories, further supporting our hypothesis [19,20]. The possible utility of MIF as a prognostic marker for CaP is suggested by the finding that serum MIF amounts are statistically increased in patients with CaP as long as 15 months post diagnosis.

Our current findings also illustrate that different protocols, or even seemingly small variations of the same protocol (such as choice of diluent), can have large effects on the ability to detect serum MIF. When we compared the same group of patients using our milk-diluent protocol (as recommended by the manufacturer) or a BSA-diluent protocol (as reported by Michael et al. [14]), we noticed that we were not able to detect differences in MIF serum levels of prostate cancer patients using the BSA-diluent protocol. This observation agrees with the findings of Michael et al. [14]. Furthermore, addition of BSA appears to diminish the ability of the ELISA protocol to detect MIF, since even the values in the same normal group were markedly smaller in the BSA-diluent protocol (reduced by 66%) when compared to our milk-diluent protocol. Thus, it is likely that the difference in the choice of diluent for the ELISA protocol accounts in part for the discrepant findings of Michael et al. [14], and their inability to detect significantly elevated MIF serum levels in their prostate cancer group. Additionally, the post-diagnosis duration was much later for our cancer group, so it is also possible that there may also be intrinsic group differences. However, the rather large discrepancy observed in a parallel test of both protocols (Fig. 2) suggests that differences in the ELISA procedure alone account for a large part of their negative findings. Particularly since MIF is documented to bind to subfractions of human albumin [17] and that albumin is a known interfering protein in serum ELISA [21], which appears to interfere with the determination of serum MIF amounts. In the aforementioned study, BSA at concentrations as high as 2.5 mg/ml was not shown to bind to immobilized recombinant MIF [17]. However, the human serum MIF ELISA as performed by both Michael et al. [14] and as reported here (Fig. 2) utilizes 4-fold higher concentrations of BSA and the MIF-BSA binding in the ELISA would occur in solution. BSA interference is a likely explanation for the discrepant findings as there were no other major differences in the serum diluent/blocking agent used including pH and ionic strength.

Interference in immunoassay is a well-documented phenomenon, especially prevalent when the analyte is detected in serum [21]. Matrix effects (defined as the sum of the effects of all of the components in a system with the exception of the analyte to be measured and including the reagents used in the assay) can increase or decrease the measured result [21]. In fact, cross-platform fluctuations in serum PSA have also been documented [22]. These interassay variations were attributed to operator variability, technical errors and/or inadequate estimation of this heterogeneous molecule and its complexes with other proteins [22,23]. All of these factors probably also affect determination of MIF serum levels and thus, strict adherence to established protocols aimed at standardization should allow for comparison of results between different laboratories.

The physiologically relevant form of MIF serum remains to be determined. However, in the serum it does not appear to be predominantly free uncomplexed MIF (monomeric, dimeric or trimeric) since these MIF forms were not detected by WB under native or denatured conditions (Fig 3A and 3B). The monomeric MIF form (12 kDa) and a small amount of the dimeric form (24 kDa) were detected in serum under reducing conditions (Fig. 3B). Crystallized recombinant bioactive MIF assumes a trimeric structure [24]. However, mixtures of all three forms of recombinant MIF have been found in solution [25]. The active *in vivo *produced MIF form(s) have not been determined. However, it was recently reported that the physiologically active form of MIF, purified from a human B-cell line was primarily dimeric (24 kDa) [26]. Based upon the results reported here the monomer, dimeric and trimeric MIF forms would account for less than 1% of the total MIF present in the serum as detected by Western blotting. In addition, our data suggest that ELISA assay conditions can alter the amount of MIF captured by the monoclonal antibody.

Interestingly, the present study also documents that majority of MIF in the serum is associated in a high molecular weight complex (180 kDa under denaturing conditions) that cannot be captured by the monoclonal antibody. We recently documented that rat MIF released by the urothelium into the bladder lumen is also part of a high molecular weight complex [27]. The rat MIF binding protein was identified as the acute phase protein α-1-inhibitor 3 [27]. α-1-inhibitor 3 is a rodent member of the α-2-macroglobulin protease inhibitor family that is found in high concentration in serum [28]. In general, the α-2-macroglobulin protease inhibitor family is recognized as carriers of a numerous cytokines and growth factors [29] and it is believed that interaction of cytokines and growth factors with α-2-macroglobulin family proteins modulates cytokine and growth factor biological activity [30]. Thus, identification of the serum complex protein may provide information about physiologically relevant MIF forms. The identities of the protein(s) in the human serum complex are presently under investigation.

The present study is the first report to document MIF mRNA expression in prostate epithelial cells and to reveal altered MIF expression in cancer using the complementary techniques of *in situ *hybridization and immunostaining within matched samples from the same patient. Low levels of MIF mRNA were restricted to the cytoplasm of prostatic epithelium in benign samples, but the MIF mRNA signal was significantly more intense in the cytoplasm of prostatic epithelial cells in matched cancer samples, suggesting that increased cytoplasmic MIF mRNA is associated with tumorigenesis. Data from our previous study (using a much smaller data set) gave some indication that increased MIF mRNA was associated with more invasive, aggressive tumor [13]. In that study, using laser capture microscopy, we reported that invasive epithelial cells exhibited higher MIF mRNA amounts than benign epithelium within the same prostatic biopsy specimen. In the present study, we demonstrate a significant increase in MIF mRNA associated with prostatic tumors compared with matched benign tissue from the same patients, documenting that upregulation of MIF gene expression is a hallmark of prostatic tumorigenesis (Fig. 1A).

This report demonstrates that MIF protein is localized to both the malignant and matched histologically benign epithelia in prostate cancer patients. This observation confirms our earlier findings [12], as well as those of other investigators [31,32]. del Vecchio et. al determined a decrease in MIF immunostaining in high grade tumors [31]. These authors suggested that this finding may be the consequence of a reduced MIF synthesis or of an enhanced and altered secretion by tumor cells into the surrounding stroma [31]. However, these studies localized and quantified MIF protein only. The alternative explanation for reduced MIF immunostaining is supported by the data presented here, namely, there is increased MIF secretion by aggressive prostate cancer cells. This hypothesis is based upon two separate sets of findings: (1) increased mRNA expression in prostate biopsy samples without a concomitant increase in cytoplasmic protein in prostate cancer epithelium suggests that tumor cells may secrete more MIF and (2) documented increased MIF secretion by prostate cancer cell lines compared with normal prostate epithelial cells in culture. We recognize that there is no correlation between MIF immunohistochemistry and serum levels. In fact, this anomaly is described for serum PSA versus PSA immunohistochemistry [33]. Based upon these findings it is tempting to speculate that the increased MIF secretion by prostate cancer cells is responsible for the increased serum MIF levels observed in prostate cancer patients. Prostate cancer disrupts acinar structure and function resulting in "leakage" of proteins normally released into the acinar ducts (e.g. PSA), into the stroma and then absorption into the circulation [34]. It is possible that a similar mechanism accounts for the increased MIF secretion by prostate cancer epithelial cells and increased MIF serum levels in cancer patients.

CD74 was identified as the receptor for MIF using *in vitro *models [11]. The primary function of this protein is to regulate peptide loading of exogenously derived peptides onto major histocompatibility class II heterodimers, but a small portion of the total cell CD74 content is expressed on the cell surface [35]. However, neither the exact function of the cell surface CD74, nor the physiological relevance of the interaction of CD74 with MIF is known [11,36]. We recently documented that MIF associates with CD74 and CD44 in an urothelial cancer cell line [10] and in an *in vivo *model of bladder inflammation [15] suggesting that the effects of MIF on the bladder are mediated by interaction with both CD74 and CD44. If MIF-CD74 interaction is needed for MIF to affect prostate tumor growth then CD74 should be localized within the prostate. To our knowledge, ours is the first report to show that CD74 is present in human prostate tissue. However, these data do not show any significant change in CD74 amount in tumor compared to matched benign prostate. Thus, the physiological relevance of CD74 in prostate cancer remains to be determined.

## Conclusion

In summary, we confirmed and extended our observations of increased serum MIF and MIF mRNA are associated with CaP. Serum MIF is predominantly part of a complex with other proteins that undetected by ELISA using a commercially available MIF mAb. MIF mRNA was observed in the cytoplasm of all prostatic epithelia, but was increased in matched cancer samples. In addition, this study documents the location of CD74, the MIF receptor, within prostate epithelial cells. Whether increased serum MIF has predictive value that is independent of known predictive markers such as rising PSA values or Gleason score in prostate cancer awaits a larger trial that compares these variables prospectively with PSA levels and patient outcomes.

## Competing interests

The author(s) declare that they have no competing interests.

## Authors' contributions

KLMS participated in the design of the study, ELISA, Western blotting, immunoprecipitations, statistical analyses, performed *in situ *hybridizations and carried out all cell culture studies. KAL photographed all tissue slides, scored all *in situ *hybridizations and immunohistochemical slides. PLV participated in the design of the study, ELISA, Western blotting, immunohistochemical analysis and statistical analyses. All authors read and approved the final manuscript.

## Pre-publication history

The pre-publication history for this paper can be accessed here:
